# Dampening of Submesoscale Currents by Air-Sea Stress Coupling in the Californian Upwelling System

**DOI:** 10.1038/s41598-018-31602-3

**Published:** 2018-09-06

**Authors:** Lionel Renault, James C. McWilliams, Jonathan Gula

**Affiliations:** 1 LEGOS, Université de Toulouse, IRD, CNRS, CNES, UPS, Toulouse, France; 2 Department of Atmospheric and Oceanic Sciences, University of California, Los Angeles, California USA; 3 Laboratoire d’Océanographie Physique et Spatiale, Université de Brest, CNRS, IRD, Ifremer, IUEM, Brest, France

## Abstract

Oceanic submesoscale currents (SMCs) occur on an scale of 0.1–10 km horizontally and have a large influence on the oceanic variability and on ecosystems. At the mesoscale (10–250 km), oceanic thermal and current feedbacks are known to have a significant influence on the atmosphere and on oceanic dynamics. However, air-sea interactions at the submesoscale are not well known because the small size of SMCs presents observational and simulation barriers. Using high-resolution coupled oceanic and atmospheric models for the Central California region during the upwelling season, we show that the current feedback acting through the surface stress dominates the thermal feedback effect on the ocean and dampens the SMC variability by ≈17% ± 4%. As for the mesoscale, the current feedback induces an ocean sink of energy at the SMCs and a source of atmospheric energy that is related to induced Ekman pumping velocities. However, those additional vertical velocities also cause an increase of the injection of energy by baroclinic conversion into the SMCs, partially counteracting the sink of energy by the stress coupling. These stress coupling effects have important implications in understanding SMC variability and its links with the atmosphere and should be tested in other regions.

## Introduction

The California Current System (CCS) is an eastern boundary upwelling system and is among the biologically most productive coastal environments^1^. As for the other eastern boundary upwelling systems, the CCS is characterized by the presence of an upwelling favorable equatorward wind during spring and summer^2,3^ that brings deep cold and nutrient-rich water to the surface, maintaining high rates of productivity over broad scales^4^. Additionally, coastal currents and oceanic mesoscale variability lead to large cross-shore exchange of heat, salt and biogeochemical tracers between the open and coastal oceans as well as between the surface layer and interior^5–7^.

The CCS is also under a strong influence of the submesoscale activity^8–10^. Oceanic submesoscale currents (SMCs) occur on an intermediate scale on the order of 0.1–10 km horizontally, *i.e*. smaller than the mesoscale currents but still large enough that rotation and density stratification matter. In the open ocean SMCs typically occur at the oceanic surface and bottom and emerge from various mechanisms such as frontal instabilities, frontogenesis, or topographic wakes^8–14^. SMCs can provide a major route of energy dissipation^15,16^. By inducing large vertical velocities, they also lead to significant vertical eddy fluxes of materials with an important influence on ecosystems^8,17,18^.

Air-sea interactions have a strong influence on the mesoscale activity and on the primary production of the CCS, in particular during the upwelling season^7,19–22^. Air-sea interactions are mainly driven by two different feedbacks from the ocean to the atmosphere: the Thermal FeedBack (TFB) and the Current FeedBack (CFB). The TFB corresponds to the impact of ocean surface temperature gradients on the atmospheric winds and stresses through a modification of the properties of the atmospheric boundary layer and associated vertical momentum fluxes^23^. The CFB corresponds to the effect of surface ocean currents in the calculation of the wind stress^24^. Both feedbacks have been shown to impact the ocean dynamics at the mesoscale. In the CCS, the TFB can influence the slackening of the wind toward the coast (wind drop-off) and, thus, the generation of eddies by baroclinic instability^2,3,7,25^. By generating fine scale wind curl anomalies, the TFB can affect eddy propagation^21^ but does not directly dampens the eddies^26^. The CFB systematically induces fine-scale structures in the surface stress^24,27^. In contrast with the TFB, these stress anomalies drastically dampens the mesoscale activity^22,27–32^. For the CCS, the eddy kinetic energy at the mesoscale is reduced by 40% when the CFB is taken into account^21,22^. This eddy dampening is mainly driven by an “eddy killing” effect, *i.e*. a sink of energy from the oceanic geostrophic currents to the atmosphere^22,30,33,34^. The resulting coupling coefficient between current vorticity and surface stress curl (*s*_*τ*_ [N s m^−3^]) expresses that the CFB acts as a sink of ocean eddy energy and a source of atmospheric energy. From a mechanistic point of view, the sink of energy is associated with Ekman pumping vertical velocities in the ocean induced by the surface stress gradients. This additional Ekman pumping, which does not significantly affect the mesoscale baroclinic instability, can be interpreted as the mechanism for weakening an eddy^28,32,35^.

**Figure 1:**
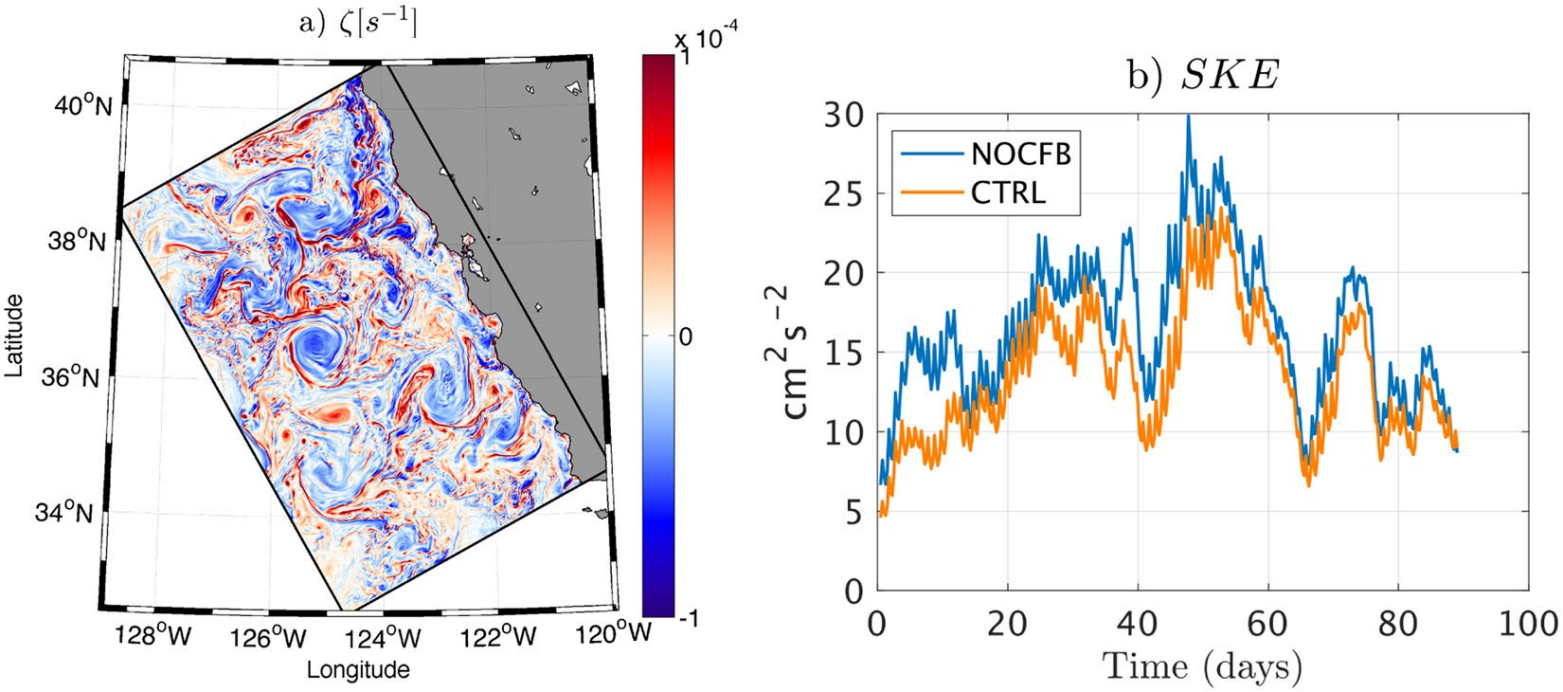
The CFB to the atmosphere causes a dampening of the submesoscale activity by roughly 17%. (**a**) Ocean (OCE) and Atmosphere (ATM) models configuration illustrated by a snapshot of surface vorticity (*ζ*) from the CTRL simulation. (**b**) Temporal evolution of the surface Submesoscale Kinetic Energy (SKE) averaged over the whole domain.

So far, relatively little is known about air-sea coupling at the submesoscale because of computational costs and measurement limitations. In particular, it is not yet known to what extent the CFB might dampen the SMCs. The focus of this study is on the characterization of the CFB effect on the SMCs variability during the upwelling season in the CCS. Specifically, we aim to answer to the following two questions: (i) What is the effect of the CFB on the SMCs during the upwelling season? (ii) What are the main related processes? To this end, the Regional Oceanic Modeling System (ROMS^36^) in its Coastal and Regional Ocean COmmunity (CROCO) version^37^ is implemented over the U.S. West Coast and is coupled with the Weather Research Forecast (WRF^38^) model using an horizontal spatial resolution of 500 m for the ocean and 2 km for the atmosphere (Fig. 1 and SI); the coarser atmospheric resolution is a compromise between wanting higher resolution overall and the greater computational cost of WRF. We run two simulations, a control one where both TFB and CFB are taken into account (CTRL simulation) and one where only the TFB is considered (NOCFB simulation). Only a 3 months summer period corresponding to the upwelling season is considered, which is enough to obtain robust SMCs statistics, at least for the considered season^16,39,40^.

## Results

### A Dampening of the Submesoscale Variability

**Figure 2:**
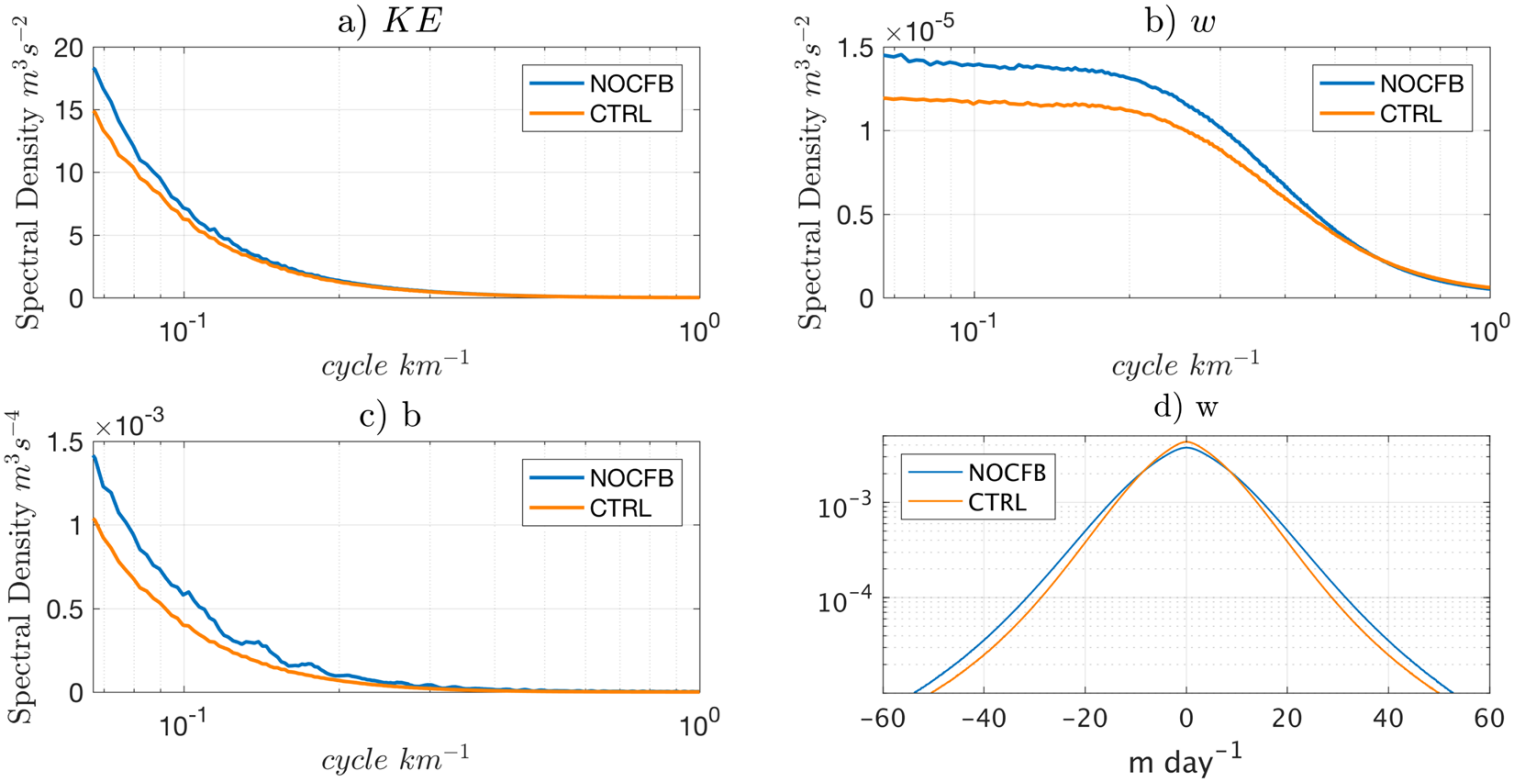
(**a**) Horizontal Surface KE spectrum, (**b**) power spectra of vertical velocity *w*, averaged between surface and 100 m depth, (**c**) power spectra of surface buoyancy *b*, (**d**) probability density function of *w* between surface and 100 m depth outside of the upwelling region to avoid the contamination by the upwelling vertical velocities (first 50 km offshore).

The CFB induces a dampening of the Submesoscale Kinetic Energy (SKE) by ≈17 ± 4% in the CCS (Fig. 1b). The surface SKE is a measure of the intensity of SMC activity, where SMC’s are computed by applying a five-point operator with a 12-km smoothing length and a 2-day smoothing interval to the currents in the simulations (as in Capet *et al*.^8^; see SI). The errorbar is obtained using a bootstrap method: the SKE difference is computed 10,000 times using random samples from the distribution. This dampening is confirmed by analyzing the surface Kinetic Energy (KE) spectrum (Fig. 2a). The spectral KE density integrated over the submesoscale range (1–12 km) is reduced by 14% in CTRL compared to NOCFB, which is close to the previous SKE reduction estimate. The SMCs are associated with large vertical velocity (*w*), horizontal vorticity (*ζ* = ∂_*x*_*v* − ∂_*y*_*u*) and horizontal buoyancy gradient at the surface^9,40^. To confirm the impact of the CFB on SMCs, the variance of *w* (from the surface to 100 m depth) and *b* are computed for NOCFB and CTRL (Fig. 2b,c). When including the CFB, both *w* and *b* variances are reduced in the submesoscale range, indicating a weakening of the submesoscale frontal intensity. The reduction of the SMCs can also be highlighted by Probability Density Functions (PDFs) for *w* and *ζ*. The PDF of *w* has a lower standard deviation with CFB. The occurrence of |*w*| > 10 m day^−1^ is reduced by 16.5% (Fig. 2d). A similar result is found for *ζ*. Finally, the weakening of the SMCs in CTRL is corroborated by a reduction of the variances of *w* from 2.14 × 10^−8^ m^2^s^−2^ to 1.76 × 10^−8^ m^2^s^−2^ (*i.e*. by 18%) and of *ζ* from 2.29 × 10^−9^ m^2^s^−2^ to 2.04 × 10^−9^ m^2^s^−2^ (*i.e*. by 13%). This indicates a general weakening of the submesoscale frontal activity when the CFB is taken into account.

**Figure 3:**
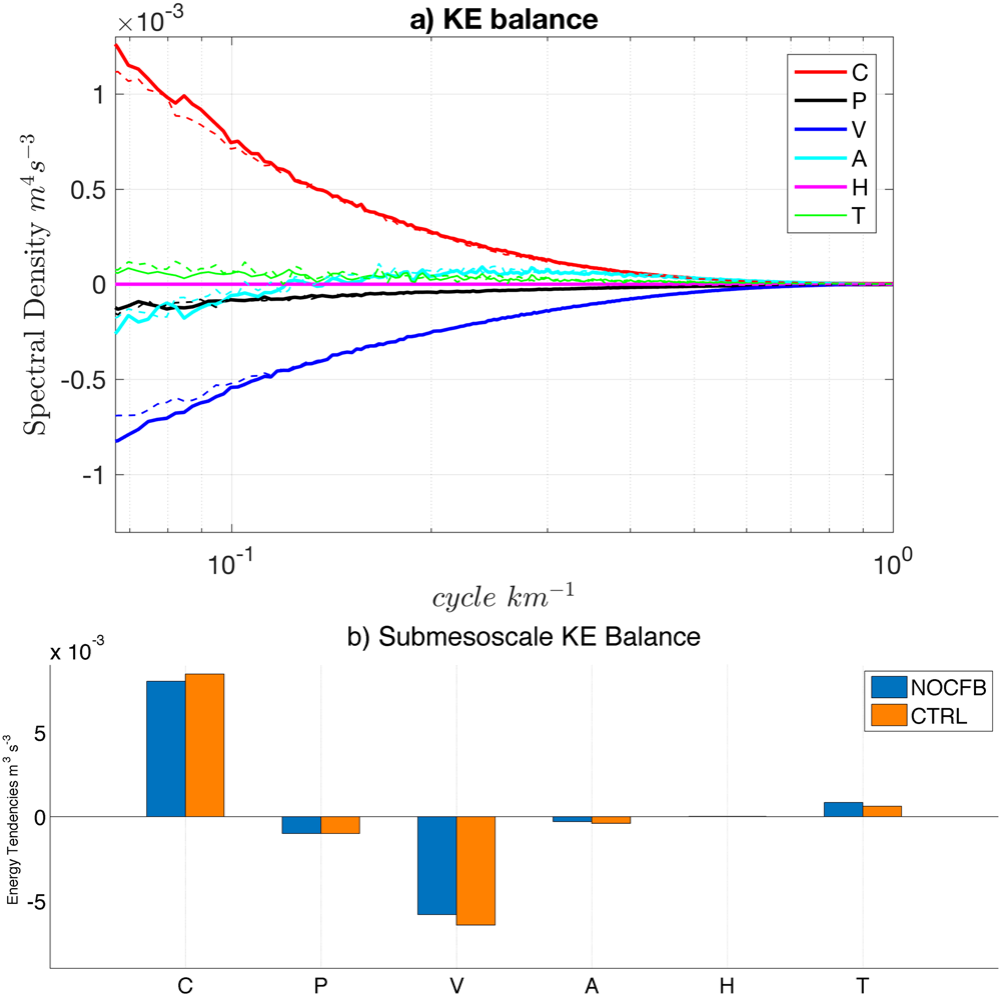
(**a**) Spectra of Kinetic Energy (KE) tendency terms [*m*^4^*s*^−3^] in the submesoscale range for injection of energy by baroclinic conversion (*C*), 3D pressure (*P*), wind and vertical mixing (*V*), 3D Advection (*A*), horizontal mixing (*H*) and the tendency term (*T*) (see SI for more details). The solid (dashed) lines represent CTRL (NOCFB). All terms have been averaged throughout the upwelling period (3 summer months) and integrated between the surface and 100 m depth. (**b**) KE tendency terms from (**a**) integrated over the submesoscale range (0.0822−0.999 *cycle km*^−1^) and between the surface and 100 m depth.

The dampening induced by the CFB is weaker at the submesoscale than at the mesoscale (*n.b*., the mesoscale Kinetic Energy dampening is ≈40%^22^). To explain it, following Capet *et al*.^10^ and Marchesiello *et al*.^41^, a spectral decomposition of the KE balance for the Primitive Equation with respect to horizontal wavenumber is computed for both simulations and averaged over the first 100 *m* (see SI and Fig. 3). Note, this balance reflects the time tendency of SKE and not its total amplitude. At the submesoscale, the KE budget is dominated by (i) an injection of energy by baroclinic conversion: *C*, *i.e*. a conversion of potential to kinetic energy due to baroclinic instability and frontogenesis; (ii) dissipation: *V*, which corresponds to the effect of the wind-work (*FK* = *τ*·***u***) and vertical mixing work. These two dominant terms are the most impacted by the CFB. *V* is enhanced by ≈10%, while surprisingly there is also more injection of energy by baroclinic conversion (*C* is ≈6% larger in CTRL than in NOCFB). The sum of the two terms is reduced when the CFB is included. The effect of the CFB on these two terms will be analyzed in the next two subsections. The nonzero tendency rate (*T*) is related to seasonal variability (only 3 months of simulations are used) and is reduced by ≈19% from NOCFB to CTRL.

### A Submesoscale Sink of Energy

**Figure 4:**
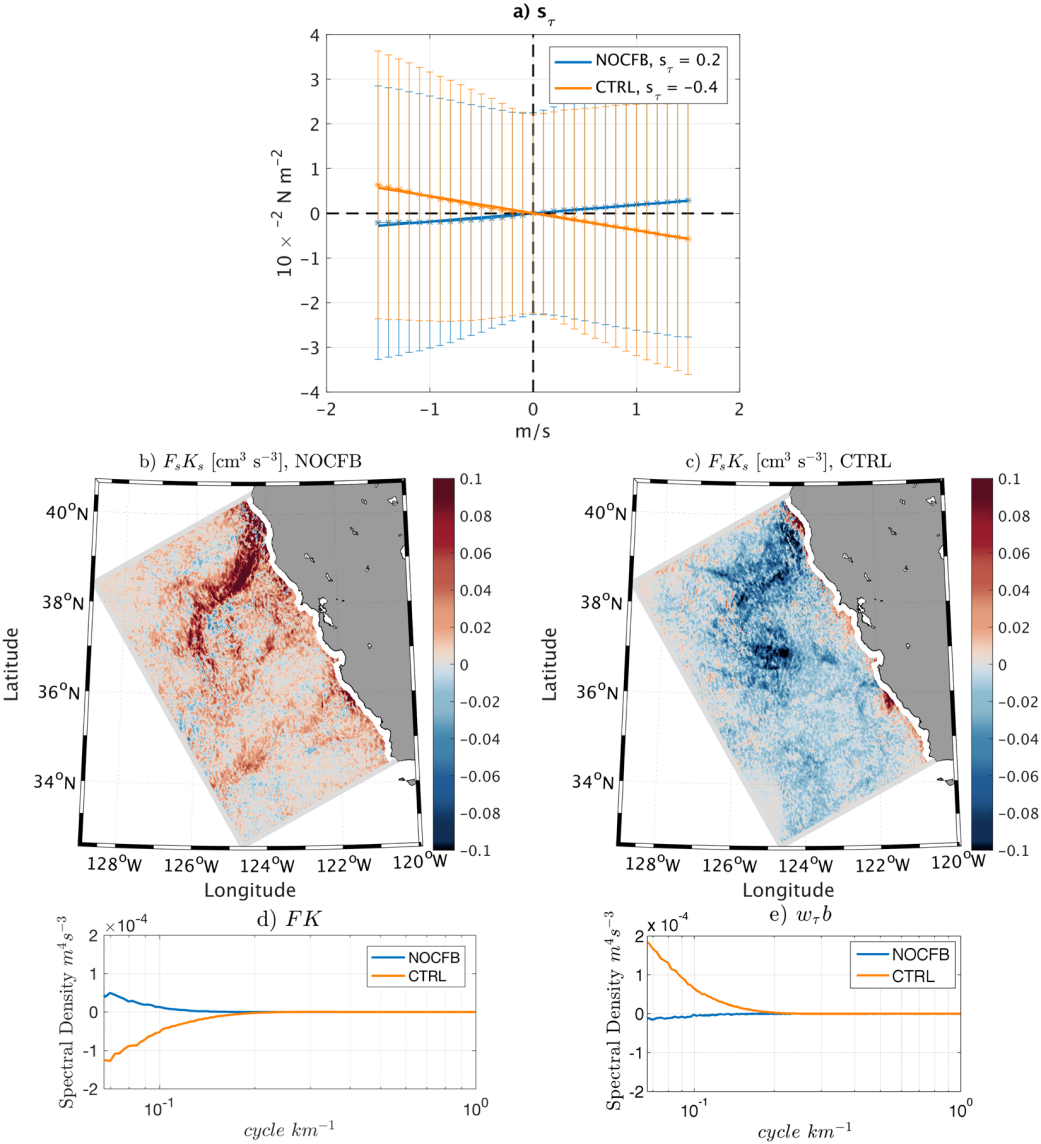
The CFB induces a sink of energy from SMCs to the atmosphere but also an additional injection of energy by baroclinic conversion that partly counteracts the sink of energy. (**a**) Binned scatter-plot of the full time series of 1-day running means of surface stress curl and surface current vorticity over the 500 m CROCO simulations domain. The bars indicate plus and minus one standard deviation from the average value (stars). The linear regression is indicated by a line and the slope *s*_*τ*_ is indicated in the legend (10^−2^ N s m^−3^). NOCFB and CTRL are represented in blue and orange, respectively. NOCFB has a slight positive slope that may be due to the TFB to the atmosphere, but the CFB overwhelms it in CTRL, causing a negative slope. The CFB induces submesoscale surface stress structures. (**b**) Mean submesoscale wind-work (*F*_*s*_*K*_*s*_) from NOCFB and (**c**) CTRL. The CFB induces a sink of energy from the SMCs to the atmosphere, which in turn dampens the SMCs. (**d**) Two-dimensional wind work (*FK* = *τ*·***u***) spectrum from NOCFB (blue) and CTRL (orange). (**e**) Two-dimensional co-spectrum of Ekman pumping induced by the stress (*w*_*τ*_) and *b*, integrated over 100 m depth for NOCFB (blue) and CTRL (orange).

To express the sink of ocean energy at the SMCs induced by the CFB, we estimate the coupling coefficient between current vorticity and surface stress curl *s*_*τ*_ at the submesoscale over the whole period (3 months) using daily means (see SI). Surprisingly, when ignoring the CFB, we get *s*_*τ*_ = 0.2 × 10^−2^ N s m^−3^, which implies an energization of the SMCs. Such an energization is not present at the mesoscale and is likely the indirect effect of the TFB on the wind at the submesoscale because the thermal signature arises through correlations with the SMCs even when the CFB is not included in the model. When the CFB is included, it overwhelms the TFB. In CTRL, a negative linear relationship is found: *s*_*τ*_ = −0.4 × 10^−2^ N s m^−3^ (Fig. 4a), which implies a sink of energy from the SMCs to the atmosphere. The amplitude of the coupling coefficient is weaker at the submesoscale than at at the mesoscale^22^ (*i.e*. −1.2 × 10^−2^ N s m^−3^). A first reason is that the TFB effect partially counteracts the CFB effect at the submesoscale, while the TFB has no impact on the coupling coefficient at the mesoscale (*s*_*τ*_ ≈ 0 at the mesoscale for TFB alone^22^). A second reason is that the estimation of *s*_*τ*_ at the submesoscale is complicated by the obscuring effect of other processes, *e.g*., the presence of atmospheric inertia gravity waves that have similar spatial frequencies than the SMCs. To illustrate it, *s*_*τ*_ is also estimated using 6-hours snapshots; in this case *s*_*τ*_ = −0.2 × 10^−2^ N s m^−3^ is smaller because inertial gravity waves are not filtered out. The TFB is present in both NOCFB and CTRL, but *s*_*τ*_ is not a good metric to characterize it. O’Neill *et al*.^42^ define a thermal coupling coefficient based on the surface stress magnitude and sea surface temperature anomalies. Such a coefficient has a value of ≈1.3 × 10^−2^ N s m^−3^ at the submesoscale in both simulations, which is similar to the values found by^42^ at the mesoscale.

An energy route induced by the CFB should be present at the submesoscale given the negative *s*_*τ*_ in CTRL. The flux of energy between the atmosphere and the SMC’s due to the wind stress is the submesoscale wind-work (*F*_*s*_*K*_*s*_ [m^3^s^−3^]), which can be defined as:

1 $${F}_{s}{K}_{s}=\frac{1}{{\rho }_{0}}\,(\overline{{\tau ^{\prime} }_{x}{u^{\prime} }_{o}}+\overline{\tau {^{\prime} }_{y}\,v{^{\prime} }_{o}}),$$

where prime denotes the submesoscale part of the signal (see SI), the overbar ^–^ is the mean over the summer period, *ρ*_0_ is the oceanic surface density, *τ*_*x*_ and *τ*_*y*_ are the zonal and meridional surface stresses and *u*_*o*_ and *v*_*o*_ are the zonal and meridional surface currents. *F*_*s*_*K*_*s*_ expresses the exchange of energy between the SMCs and the atmosphere. Figure 4b,c shows *F*_*s*_*K*_*s*_ estimated from NOCFB and CTRL. Consistently with the positive *s*_*τ*_, NOCFB has a positive *F*_*s*_*K*_*s*_, *i.e*. a slight energization of the SMCs by the atmosphere induced by the TFB. At the mesoscale this TFB energization is not present. At the submesoscale the wind has not previously been identified as a significant source (or sink) of energy because of the smoothness of the stress product used to force the oceanic model^8^. When taking into account the CFB in CTRL, because of a negative *s*_*τ*_, *F*_*s*_*K*_*s*_ becomes negative, indicating a sink of energy at the SMCs and a source of atmospheric energy. At the submesoscale the CFB overwhelms the TFB effect on *F*_*s*_*K*_*s*_. The domain averaged *F*_*s*_*K*_*s*_ magnitude is indeed 3.5 times larger in CTRL than in NOCFB. It should, thus, induce a dampening of the submesoscale activity and explain the enhanced *V* in the KE balance (Fig. 3). Those results are confirmed by analyzing the wind-work (*FK* = *τ*·***u***) wavenumber spectrum. The wind-work spectrum is always positive in NOCFB, indicating a slight energization of the ocean at the submesoscale while, consistently with the previous results, the wind-work spectrum is always negative in CTRL, indicating a sink of energy at all scales. The magnitude of the power spectrum is 2 to 7 times larger in CTRL than in NOCFB, confirming the primary effect of the CFB on the wind-work. Finally, the difference in wind-work spectrum between NOCFB and CTRL explains the difference of *V* (averaged over 100 m depth) in Fig. 3, confirming its primary role in dampening the SMCs.

### A Catalizer of Submesoscale Baroclinic Instability

The increase of *C* that partly counteracts the SMCs dampening by submesoscale wind-work is less straightforward to explain. Indeed, consistently with a reduction of the SMCs, both vertical velocities and *b* are reduced in CTRL with respect to NOCFB. The increase of *C* could have been explained by a larger mesoscale Available Potential Energy (APE) reservoir available to energize SMCs (see SI). However, from NOCFB to CTRL, the APE is reduced by roughly 8% (not shown), which is consistent with a reduction of the mesoscale activity. The reduction of the APE is mechanically explained by the dampening of the mesoscale activity, which in turn causes a weakening of the mesoscale buoyancy horizontal gradients. Hence, the energization of SMCs due to submesoscale baroclinic instability should be weaker when including the CFB. However, the baroclinic conversion can also include effects related to Ekman pumping vertical velocities.

In CTRL, the additional Ekman pumping vertical velocities induced by the CFB (*w*_*τ*_ i, see SI) are responsible for the enhanced *V* and the resulting sink of energy^22,35^ but also have positive correlations with the SMCs and the buoyancy fronts. *w*_*τ*_ may therefore cause an additional injection of energy by baroclinic conversion, which is not the case at the mesoscale. The wave-number spectrum of baroclinic conversion induced by *w*_*τ*_ (*i.e. C*_*τ*_, co-spectrum of *w*_*τ*_ and *b*) is computed for NOCFB and CTRL. Without CFB, at the submesoscale, *C*_*τ*_ is slightly negative. With CFB, *C*_*τ*_ becomes positive and is even larger than the wind-work spectrum (Fig. 4d,e). For the CCS, the potential reduction of *C* due to weaker mesoscale APE is overwhelmed by the effect of *w*_*τ*_, explaining the larger injection of energy in CTRL with respect to NOCFB. It also explains why the SMCs are reduced by 17% only, which is much weaker than the reduction of the mesoscale activity (≈40%): over the CCS, the CFB induces a sink of energy from the SMCs to the atmosphere that is partly counteracted by an enhancement of the energy cascade and, thus, the baroclinic conversion.

## Discussion

In this study, we assess the impact of the Current FeedBack (CFB) on Submesoscale Currents (SMCs) using high-resolution ocean-atmosphere coupled models carried out over the California Current System (CCS) during the upwelling season. We show that the main effect of the CFB is to cause a dampening of the Submesoscale Kinetic Energy (SKE) by roughly 17%. As at the mesoscale, the CFB induces a sink of energy from SMCs to the atmosphere that also involves additional Ekman pumping in the ocean. The reduction of the mesoscale activity causes a weakening of the APE; however, the injection of energy by baroclinic conversion is not reduced, as might be expected, but rather enhanced. The sink of energy by wind-work is in fact partially counteracted by a second effect of the induced Ekman pumping: the induced vertical velocities cause an additional injection of kinetic energy into SMCs by enhanced cascade of energy and baroclinic conversion. This injection by Ekman pumping dominates over the reduction of the mesoscale APE source and partly re-energizes the SMCs relative to their wind-work depletion.

At the submesoscale, over the California Upwelling System, the coupling coefficient between SMC vorticity and surface stress (*s*_*τ*_) is positive in a simulation that considers only TFB, which is not expected at the mesoscale: a simulation that neglects the CFB has a *s*_*τ*_ ≈ 0^22^. It further induces a positive submesoscale wind-work and, thus, a slight energization of the SMCs by the atmosphere. The positiveness of *s*_*τ*_ can be explained by the TFB that induces submesoscale surface stress anomalies that are to some extent coherent with the SMCs. However, the positiveness of *s*_*τ*_ and the resulting energization of the SMCs is overwhelmed by the CFB effect, resulting in a negative *s*_*τ*_ and a sink of energy from SMCs to the atmosphere. Future studies should aim at better understanding the atmospheric response to the TFB and CFB. We also show that the effect of the TFB effect on the wind is the same with or without CFB. The TFB at the submesoscale is expected to have an impact on the turbulent heat fluxes. At the mesoscale this impact is important and may modulate western boundary currents^43,44^ and eddy propagation^44^. The TFB effect at the submesoscale should be studied using a similar approach as Seo *et al*.^44^, *i.e*. by smoothing the sea surface temperature sent to the atmospheric model by the ocean model; it may have an effect on the baroclinic conversion.

The approach used in this study has some limitations. Both TFB and CFB are considered; however, the oceanic surface waves that are neglected here (by use of the bulk formula for stress) can have other feedbacks with the ocean and the atmosphere. In particular, the Stokes vortex force^45^ can directly impact SMCs and the loss of momentum flux and energy due to wave generation^46^ can alter the exchange of energy between SMCs and the atmosphere and thus the intensity of the SMCs. In addition, the bulk formula used in this study^47^ may not be accurate at the submesoscale because it was developed mainly with attention to larger scales.

Overall, the results presented here are only valid for the CCS during the upwelling season. The CFB to the atmosphere should have an influence on the SMCs everywhere in the world ocean and for other time periods; however, the SMCs could have different local responses. For the CCS, during the upwelling season, the main drivers that explain the alteration of the SMCs variability are the mixed-layer baroclinic conversion and the negative submesoscale wind-work. During the non-upwelling season the wind is weaker than during the upwelling season and the SMCs are also slightly less energetic. As *s*_*τ*_ depends on the wind^34^, on one hand, the sink of energy by the wind work might be weaker. On the other hand, the induced Ekman pumping velocities should also be diminished, reducing the additional baroclinic instability. In other regions, the dampening of the SMCs could cause a reduction in the density restratification process caused by the submesoscale^48^, thus further induces a deepening of the mixed layer; this restratification might counteract the reduction of the mesoscale APE by the weakening of the mesoscale buoyancy horizontal gradients. Near western boundary currents, as the Gulf Stream or the Agulhas Current, in addition to the open-ocean mechanisms, SMCs can be generated by both mixed-layer baroclinic and barotropic instabilities^49^. The CFB also causes a slow-down of the western boundary currents^26,50^ and a reduction of the mean wind-work. It could in turn reduce the submesoscale activity generated by baroclinic conversion from the mesoscale eddies and boundary currents. Futures studies are needed for the SMC response to the CFB in other regions, other or longer time-period and to test the role of the spatial resolution.

## Electronic supplementary material

Supplementary Information
