# Supplementary material for: Dampening of Submesoscale Currents by Air-Sea Stress Coupling in the Californian Upwelling System
Source: Sci Rep. 2018 Sep 6;8:13388. doi: 10.1038/s41598-018-31602-3 (PMC6127151; doi:10.1038/s41598-018-31602-3)
Supplement: Supplementary file 1 — Supplementary Information [file 41598_2018_31602_MOESM1_ESM.pdf]

Supplemental Information, Dampening of Submesoscale Currents by Air-Sea Stress Coupling in the Californian Upwelling System

July 6, 2018

[1,2,*]Lionel Renault, [1]James C. McWilliams, [3]Jonathan Gula

(1) LEGOS, Universit de Toulouse, IRD, CNRS, CNES, UPS, Toulouse, France

(2) Department of Atmospheric and Oceanic Sciences, University of California, Los Angeles, California, USA

(3) Laboratoire d'Ocanographie Physique et Spatiale, Univ. Brest, CNRS, IRD, Ifremer, IUEM, Brest, France

(*) lionel.renault@ird.fr

1. Methods

The numerical models and configurations are similar to the ones employed in Renault et al. (2016b), and the following models descriptions are derived from there with minor modifications.

a. Models description

1) CROCO

The oceanic simulations were performed with the Regional Oceanic Modeling System (ROMS) (Shchepetkin and McWilliams 2005; Shchepetkin 2015) in its CROCO (Coastal and Regional Ocean Community) version (Debreu et al. 2012). CROCO is a free-surface, terrain-following coordinate model with split-explicit time stepping and with Boussinesq and hydrostatic approximations. The model is implemented in a configuration with four offline nested grids. As in Renault et al. (2016b), the coarser grid extends from 170°W to 104°W and from 18°N to 62.3°N along the U.S. West Coast and is 322 x 450 points with a resolution of 12 km. Its purpose is to force the second domain. The second domain grid extends from 144.7°W to 112.5°W and from 22.7°N to 51.1°N (Figure 1, main manuscript). The model grid is 437 x 662 points with a resolution of 4 km. Both the third and fourth domain grids encompass the Central California region. The grids are 502 x 762 points with a resolution of 1 km and 1000 x 1520 points with a resolution of 500 m, respectively. As in Capet et al. (2008a), the assumption underlying this approach is that SMCs developing at higher resolutions do not have significant upscaling effects on the mesoscale or mean circulation outside the Central California domain. The models have a similar configuration to Renault et al. (2016b), with 42 vertical levels for the 4 km and 1 km grids, and 60 for the 500 m grid; the vertical grid is stretched for increased boundary layer resolution using stretching surface and bottom parameters of $h_{cline} = 250\ m$, $\theta_b = 1.5$, and $\theta_s = 6.5$.

Initial and horizontal boundary data for T, S, surface elevation, and horizontal velocity are taken from the quarter-degree, daily-averaged Mercator Glorys2V3 product (http://www.myocean.eu). The 12 km and the 4 km domains are run for 5.5 years from June 1995 to June 2000, using interannual lateral oceanic and surface forcing. The 1 km and the 500 m domains are run from June to September 2000. Only the summer period (July-September) is analyzed. Vertical mixing of tracers and momentum is done with a K-Profile Parameterization (KPP; Large et al. 1994). The diffusive part of the advection scheme is rotated along the isopycnal surfaces to avoid spurious diapycnal mixing (Lemarié et al. 2012).

The atmospheric fields are simulated using the Weather Research and Forecast (WRF) model. The Fairall et al. (2003) bulk formulae is used to estimate the freshwater, turbulent, and momentum fluxes provided to the ocean model. Two sets of simulations are done. In the first set (NOCFB4, NOCFB1, NOCFB500), WRF gives the ocean model hourly averages of freshwater, heat, and momentum fluxes; whereas, the ocean model sends WRF the hourly SST. In the second set (CFB4, CFB1, CFB500), the ocean model sends WRF both SST and surface currents,, and the surface stress is estimated using the relative wind to the ocean motions:

$U_r = U_a - U_o$. Note that the use of relative winds also involves a modification of both the surface-layer vertical mixing parameterization (MYNN2.5 in our case) and the tridiagonal matrix for vertical turbulent diffusion Lemarié (2015).

2) WRF

WRF (version 3.7.1, Skamarock et al. 2008) is implemented in a configuration with three grids. The domains is slightly larger than the ocean domains to avoid the effect of the WRF sponge (4 points). They have a horizontal resolution of 18 km, 6 km, and 2 km. The 18 km and 6 km domains are initialized with the Climate Forecast System Reanalysis (CFSR) ($\approx$ 40 km spatial resolution; Saha et al. 2010) from 30th December 1994 and integrated for 5.5 years with time-dependent boundary conditions interpolated from the same six-hourly reanalysis. Forty vertical levels are used, with half of them in the lowest 1.5 km, as in Renault et al. (2016a). The parameterizations used are the same as Renault et al. (2016b). Note WRF has a slight coarser spatial resolution than the ocean model mainly because of the computational cost, which is much higher for an atmospheric model. Moreover, when coupling a 500-m oceanic model, having a 2-km atmospheric model is enough because (1) as shown by Renault et al. (2017), s_τ depends on the large scale wind, and (2) in the atmospheric model, the Planet Boundary Layer (MYNN2.5) response to the current and thermal feedbacks is basically 1D and the CROCO effective resolution is about $4\delta x$ (*i.e.,* $\approx 2.5km$ for a 500m solution), thus, a 2-km resolution in the atmospheric model is enough to reproduce the atmospheric response to the current and thermal feedbacks.

b. Current Feedback in a Coupled Model

In a coupled model the current feedback to the atmosphere is simply represented by using a bulk formulae for stress with the surface wind relative to the oceanic current:

$$\boldsymbol{U_r} = \boldsymbol{U_a} - \boldsymbol{U_o}\,, \tag{1}$$

where $\boldsymbol{U_a}$ and $\boldsymbol{U_o}$ are the surface wind and the surface current at the closest model grid levels to the surface, respectively. When neglecting the current feedback, the wind U_a is used instead of the relative wind U_r. As described by Lemarié (2015), because of the implicit treatment of the bottom boundary condition in most atmospheric models, the use of relative winds involves a modification of both the surface-layer vertical mixing parameterization (MYNN2.5 in our case) and the tridiagonal matrix for vertical turbulent diffusion.

c. *Submesoscale characterization*

As in Capet et al. (2008a), the submesoscale is isolated from the signal using a high-pass spatial filter that consists of a five-point operator with a 12-km smoothing length and 2-days smoothing. Note, the use or not of the 2-days smoothing does not change qualitatively the results.

d. *Spectrum*

To compute the spectrum the area mean is first removed and a symmetrization is performed using an Hanning window Jenkins and Watts (1968).

e. *KE budget*

Following Capet et al. (2008b) and Marchesiello et al. (2011), a spectral decomposition of the KE balance for the Primitive Equations has been computed. To that end, the co-spectrum between velocity and the term that enters the momentum equations is computed on each 6-hours snapshots and, then, averaged over the 3 months-period considered here and over 100m depth (note similar results are found when considering only *e.g.,* 70m depth). It is expressed as:

$$T(k) = C(k) + P(k) + V(k) + A(k) + R(k) \tag{2}$$

$$C(k) = \mathbb{R}\left[\overline{\widehat{w}^{*}b}\right] \tag{3}$$

$$P(k) = \mathbb{R}\left[\overline{[-\frac{1}{\rho_0}\widehat{\boldsymbol{u}}^{*} \cdot \widehat{\nabla p}}\right] \tag{4}$$

$$V(k) = \mathbb{R}\left[\overline{\widehat{\boldsymbol{u}_h^{*}} \cdot \frac{\partial \widehat{K_v \frac{\partial \boldsymbol{u}_h}{\partial z}}}{\partial z}}\right] \tag{5}$$

$$A(k) = \mathbb{R}\left[\overline{-\widehat{\boldsymbol{u}_h^{*}} \cdot (\boldsymbol{u}_h \cdot \widehat{\nabla})\boldsymbol{u}_h - \widehat{\boldsymbol{u}_h^{*}} \cdot w\frac{\widehat{\partial \boldsymbol{u}_h}}{\partial z}}\right] \tag{6}$$

where b is buoyancy ($b = -g\frac{\rho}{\rho_0}$) and K_v is the vertical viscosity. The caret stands for an horizontal Fourier transform after removing the area mean and applying a 2D Hanning window Jenkins and Watts (1968), which has the effect to symmetrize the signal and suppressing the advective horizontal fluxes through

the boundary. * denotes the complex conjugate operator; $\mathbb{R}$ represents the real part operator; — indicates an average over the 3-months considered here and over 100m depth, (at 100m depth the vertical flux is small, choosing 70m depth does not qualitatively change the results). C represents an injection of energy by baroclinic conversion; P is the 3D pressure work; V corresponds to the wind and vertical mixing work; A represents the horizontal and vertical advection contribution; and the horizontal mixing (H).

f. Coupling Coefficient s_τ

In this study the submesoscale s_τ is defined as the linear regression between submesoscale surface stress curl and oceanic current vorticity and is evaluated over the 500 m domain and over the summer 2000. The fields are first temporally averaged using a 1-day running mean.

g. Available Potential Energy

The mesoscale APE is estimated as Fox-Kemper et al. (2008); Srinivasan et al. (2017):

$$APE = \frac{1}{2} H_b^2 \overline{|\nabla_h b_{ME}|}^z, \tag{7}$$

where b_{ME} represents the mesoscale component estimated using a spatial low-pass filter of 250 km, and the depth average is computed over the mixed layer depth H_b. H_b is estimated using the classic approach of the depth at which the density is 0.03 °C below that at 10 m depth (de Boyer Montégut et al. (2004)).

h. Surface Stress Induced Ekman Pumping

The Ekman pumping induced by the surface stress is computed as:

$$w_\tau = \boldsymbol{k} \cdot \nabla \times \frac{\boldsymbol{\tau}}{\rho_0 f}\,, \tag{8}$$

where f is the Coriolis frequency.

References

Capet, X., McWilliams, J. C., Molemaker, M. J., and Shchepetkin, A., 2008a: Mesoscale to submesoscale transition in the California Current System. Part I: Flow structure, eddy flux, and observational tests.

Journal of Physical Oceanography, **38(1)**, 29–43.

Capet, X., McWilliams, J. C., Molemaker, M. J., and Shchepetkin, A., 2008b: Mesoscale to submesoscale transition in the California Current System. Part III: Energy balance and flux. *Journal of Physical Oceanography*, **38(10)**, 2256–2269.

de Boyer Montégut, C., Madec, G., Fischer, A. S., Lazar, A., and Iudicone, D., 2004: Mixed layer depth over the global ocean: An examination of profile data and a profile-based climatology. *Journal of Geophysical Research: Oceans*, **109(C12)**.

Debreu, L., Marchesiello, P., Penven, P., and Cambon, G., 2012: Two-way nesting in split-explicit ocean models: Algorithms, implementation and validation. *Ocean Modelling*, **49**, 1–21.

Fairall, C., Bradley, E. F., Hare, J., Grachev, A., and Edson, J., 2003: Bulk parameterization of air-sea fluxes: Updates and verification for the COARE algorithm. *Journal of Climate*, **16(4)**, 571–591.

Fox-Kemper, B., Ferrari, R., and Hallberg, R., 2008: Parameterization of mixed layer eddies. Part I: Theory and diagnosis. *Journal of Physical Oceanography*, **38(6)**, 1145–1165.

Jenkins, G. M., and Watts, D. G., 1968: Spectral analysis.

Large, W. G., McWilliams, J. C., and Doney, S. C., 1994: Oceanic vertical mixing: A review and a model with a nonlocal boundary layer parameterization. *Reviews of Geophysics*, **32(4)**, 363–404.

Lemarié, F., 2015: Numerical modification of atmospheric models to include the feedback of oceanic currents on air-sea fluxes in ocean-atmosphere coupled models. Technical Report RT-464, INRIA Grenoble - Rhône-Alpes ; Laboratoire Jean Kuntzmann ; Universite de Grenoble I - Joseph Fourier ; INRIA.

Lemarié, F., Kurian, J., Shchepetkin, A. F., Molemaker, M. J., Colas, F., and McWilliams, J. C., 2012: Are there inescapable issues prohibiting the use of terrain-following coordinates in climate models? *Ocean Modelling*, **42**, 57–79.

Marchesiello, P., Capet, X., Menkes, C., and Kennan, S. C., 2011: Submesoscale dynamics in tropical instability waves. *Ocean Modelling*, **39(1)**, 31–46.

Renault, L., Hall, A., and McWilliams, J. C., 2016a: Orographic shaping of U.S. West Coast wind profiles during the upwelling season. *Climate Dynamics*, 1–17.

Renault, L., McWilliams, J. C., and Masson, S., 2017: Satellite Observations of Imprint of Oceanic Current on Wind Stress by Air-Sea Coupling. *Scientific Reports*, **7(1)**, 17747.

Renault, L., Molemaker, M. J., Mcwilliams, J. C., Shchepetkin, A. F., Lemarié, F., Chelton, D., Illig, S., and Hall, A., 2016b: Modulation of Wind Work by Oceanic Current Interaction with the Atmosphere. *Journal of Physical Oceanography*, **46(6)**, 1685–1704.

Saha, S., Moorthi, S., Pan, H.-L., Wu, X., Wang, J., Nadiga, S., Tripp, P., Kistler, R., Woollen, J., Behringer, D., et al., 2010: The NCEP climate forecast system reanalysis. *Bulletin of the American Meteorological Society*, **91(8)**, 1015–1057.

Shchepetkin, A. F., 2015: An adaptive, Courant-number-dependent implicit scheme for vertical advection in oceanic modeling. *Ocean Modelling*, **91**, 38–69.

Shchepetkin, A. F., and McWilliams, J. C., 2005: The Regional Oceanic Modeling System (ROMS): A split-explicit, free-surface, topography-following-coordinate oceanic model. *Ocean Modelling*, **9(4)**, 347–404.

Skamarock, W., Klemp, J., Dudhia, J., Gill, D., and Barker, D., 2008: A description of the Advanced Research WRF version 3. NCAR. Tech. rep., Note NCAR/TN-4751STR.

Srinivasan, K., McWilliams, J. C., Renault, L., Hristova, H. G., Molemaker, J., and Kessler, W. S., 2017: Topographic and mixed layer submesoscale currents in the near-surface Southwestern Tropical Pacific. *Journal of Physical Oceanography*, **47(6)**, 1221–1242.
